# Would the choice of multiplex platform impact the management of the allergic patient? A first approach focusing on LTPs

**DOI:** 10.1002/jcla.24960

**Published:** 2023-08-28

**Authors:** Carmen Maria Cabrera, Moisés Labrador Horrillo, Francisco Feo Brito, Alberto Palacios‐Cañas

**Affiliations:** ^1^ Allergy and Immunology Section Ciudad Real University General Hospital Ciudad Real Spain; ^2^ Faculty of Medicine of Ciudad Real University of Castilla‐La Mancha Ciudad Real Spain; ^3^ Allergy Section Vall d'Hebron University Hospital Barcelona Spain; ^4^ Autonomous University of Barcelona Barcelona Spain; ^5^ Allergy Section Ciudad Real University General Hospital Ciudad Real Spain

**Keywords:** ALEX2, clinical impact, comparison, ISAC, LTP

## Abstract

**Background:**

In the Mediterranean area, patients with LTP syndrome who are sensitized to multiple allergens are often tested for sIgE using multiplex platforms. The results obtained from different commercial platforms are not interchangeable, so it is important to compare and validate the platform selected for use. The objective of this study is to compare and validate the performance of the ImmunoCAP ISAC E112i and the macroarray ALEX2 in our daily practice.

**Methods:**

From August 2021 to March 2022, we tested 20 random serum samples from polysensitized patients using the ALEX2 test (MADx) and ImmunoCAP tIgE and ISAC E112i (Thermo Fisher Scientific). We compared the total IgE (tIgE) and sIgE levels for shared allergens.

**Results:**

The heatmap generally showed more intense results for ISAC. The overall correlation was good, but some exceptions were noted. The main discrepancies were found for Ole e 7, which was positive for 11 patients in ISAC but negative for all patients in ALEX2, and for nut LTPs, for which ISAC showed a threefold higher detection rate for Ara h 9 and a fivefold higher detection rate for Cor a 8 and Jug r 3 compared to ALEX2. The regression model showed no interchangeability of tIgE results.

**Conclusions:**

Despite our small sample size and the complexity of comparing a quantitative and a semi‐quantitative platform, our results suggest that patient diagnosis and management can be influenced by the platform used. Therefore, our findings must be taken into consideration when choosing a platform to use for some profiles of LTP‐polysensitized patients, even though more data is needed.

## 1 BACKGROUND

In our catchment area, Ciudad Real province, we manage a high number of complex polysensitized patients, many of whom are diagnosed with lipid transfer proteins (LTP) syndrome, a typical Mediterranean pattern.^1^ We generally use multiplex testing to study these patients.

The 2 main multiplex sIgE determination platforms in use today are the microarray ImmunoCAP ISAC E112i and the macroarray ALEX2.^2^ Given that the results generated are not interchangeable between the platforms,^3^ it is important to clinically validate both methods.

Several studies have compared ALEX2 with ISAC, although extensive clinical validation is still an unmet need.^4^, ^5^ Moreover, analysis of published data reveals discrepancies: Scala et al^6^ found that ISAC performed better for panallergens, whereas Quan et al^5^ concluded that the results of both methods are comparable.

The aim of our study was to compare the agreement between clinical symptoms and sensitization profiles revealed by ALEX2 and ISAC when used in daily clinical management of polysensitized patients, with emphasis on sensitization to LTPs. However, given the complexity of the patients and the high number of allergens covered by both platforms, we started by comparing the results of both assays for 20 patients to determine where to focus our efforts in a validation study using a homogeneous cohort.

## 2 METHODS

The study included 20 routine serum samples from polysensitized patients referred for multiplex testing from August 2021 to March 2022, with suspected LTP syndrome based on compatible food and/or pollen clinical allergy, sIgE and/or prick test results. Both adults and children were included. All patients were tested using the ALEX2 test (MADx) and ImmunoCAP tIgE and ISAC E112i (Thermo Fisher Scientific), Total IgE (tIgE) and sIgE load for shared allergens were compared. The assays were run according to the manufacturer's instructions. (Appendix S1 for detailed inclusion criteria and statistical analysis).

## 3 RESULTS

The mean age of the 20 study patients was 29 years (range, 6–54 years). Patients were evenly distributed by sex. Sensitization and clinical profile details are described in Tables S1 and S2.

**FIGURE 1:**
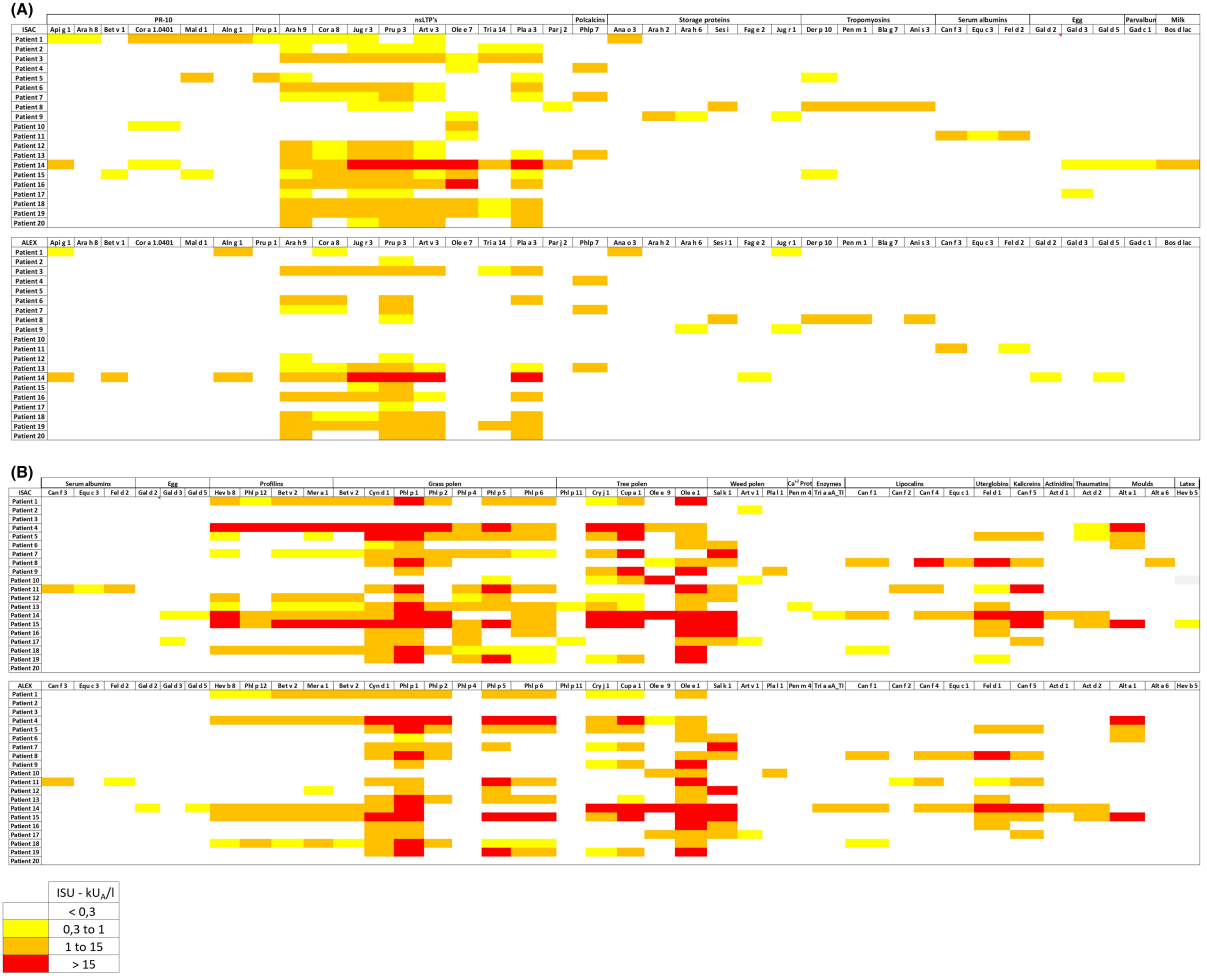
(A) Heatmap corresponding to the sensitization profiles of the 20 study patients according to ALEX and ImmunoCAP ISAC. The heatmap displays only allergens shared between both platforms with at least a positive result in either of the two platforms. Allergens are grouped by protein families. (B) Heatmap corresponding to the sensitization profiles of the 20 study patients according to ALEX and ImmunoCAP ISAC. The heatmap displays only allergens shared between both platforms with at least a positive result in either of the two platforms. Allergens are grouped by protein families.

In general, the heatmap showed more intense results for ISAC. We also observed that some shared LTPs were positive in ISAC and negative in ALEX2, whereas no positive ALEX2 result corresponded to a negative ISAC result. The greatest difference in the heatmap pattern was displayed by the panallergens (Figure 1).

Considering a quantitative comparison for both methods, the overall correlation was good, with lower agreement recorded for Jug r 3, Tri a 14, and Par j 2. No agreement was observed for Ole e 7, as no results were shown to be positive by ALEX2 (Figures S1 and S2).

Fourteen (70%) patients were positive for Pru p 3 by both platforms. For pollen LTPs, ISAC revealed that 11 of those patients were sensitized to Ole e 7 (55%) (Table S2), although the results were negative for the “research use only” Ole e 7 included in ALEX2. Interestingly, 5 patients (25%) had sensitization loads to Ole e 7 that were equivalent to those of Ole e 1 and/or Ole e 9, or clearly higher, with olive pollen being the main sensitizer in 2 of them (10%) (Table S3). The 5 patients had a clinical diagnosis of olive pollen allergy.

Fifty percent of the patients (*n* = 10) experienced clinical symptoms with nuts. According to clinical records, six individuals showed clinically relevant Ara h 9 sensitization, one to Cor h 8, and four to Jug r 3. ALEX2 reported 2 Jug r 3 and 2 Ara h 9 false negative results, respectively. ISAC reported 1 Ara h 9 false negative result. Average ALEX2 values for Ara h 9 and Jug r 3 were 59% and 81% lower than ISAC values, respectively. (Table S2b). The analysis considering just sensitization status is shown in Table S2a. Three out of ten patients had sensitization to storage proteins at equal or higher loads than LTPs. They all were detected by both platforms.

Although 4 patients were sensitized to Sola l 6 (tomato 7 kDa‐LTP) in ALEX2, the results were negative for the whole extract in 3 cases (75%) (Table S4).

The regression model used in the tIgE interchangeability evaluation yielded an r^2^ of 0.559, indicating no clear trend for market variability. For some patients, ImmunoCAP yielded higher results for total IgE; however, we recorded contrasting results for other patients (Figures S3 and S4).

## 4 DISCUSSION

Although preliminary, our results highlight important details to consider when analyzing patient sensitization profiles with both platforms.

We observed that ALEX2 Ole e 7 did not work well in the study population: olive pollen was the main tree pollen sensitizer, as reported elsewhere.^5^, ^6^ Ole e 7 revealed relevant sensitization in 25% of patients. This result is clinically relevant in areas with high olive pollen pressure since olive pollen immunotherapy is not recommended in this type of patient.^7^

When focusing on nut LTPs, we found that ALEX2 performed more poorly, especially for Jug r 3, the second most important LTP in the Mediterranean area.^8^ Although the Spearman correlation was good, we saw that while Jug r 3 was clearly working better for patients with high sensitization levels, it failed in patients with low sensitization levels or with high sensitization levels but low tIgE, thus suggesting low sensitivity for these components, as reported previously for Ara h 9 and Cor a 8.^9^ In fact, 3 of the patients who were positive for ISAC Jug r 3 and negative for ALEX2 had tIgE levels below 100 kU/L. This finding is also clinically relevant, as the therapeutic approach for storage proteins and LTPs differs depending on whether they are displayed alone or in combination (Tables S2a and S2b).

Finally, we noticed that Sola l 6 seemed to capture most tomato sIgE, leaving few free sIgE to be detected by the whole extract, indicating that sIgE to the extract and the component are competing against each other. However, this behavior can also be related to an underrepresentation of Sola l 6 in the whole extract. To date, more studies are needed to understand the competition between whole extracts and the corresponding components in multiplex platforms.

A poor correlation was found for total IgE measured by ALEX and ImmunoCAP. We used ImmunoCAP for total IgE because ISAC cannot determine total IgE, thus showing that total IgE results are not interchangeable with the gold standard ImmunoCAP and that probably total IgE by ALEX2 is not an adequate basis on which to calculate ratios. However, it needs to be further evaluated in clinical studies.

## 5 CONCLUSIONS

Despite our small sample size, and the fact that a quantitative and a semiquantitative platforms comparison is complex our results suggest that patient diagnosis and management can be affected by the platform used, and although more data are needed, our findings must be considered when deciding which platform to use in some profiles of LTP‐polysensitized patients.

## FUNDING INFORMATION

Thermo Fisher Scientific provided free‐of‐charge ImmunoCAP ISAC e112i tests.

## CONFLICT OF INTEREST STATEMENT

The authors declare no conflicts of interest.

## Supporting information


Appendix S1.

## Data Availability

Tha data that support the findings of this study are available on request from the corresponding author. The data are not publicly available due to the privacy or ethical restrictions.
