# Supplementary material for: Would the choice of multiplex platform impact the management of the allergic patient? A first approach focusing on LTPs
Source: J Clin Lab Anal. 2023 Aug 28;37(15-16):e24960. doi: 10.1002/jcla.24960 (PMC10561593; doi:10.1002/jcla.24960)

**S1. Inclusion Criteria**

- Patients with clinical allergy, including food allergy and allergic asthma or allergic rhinitis, diagnosed by an allergy specialist.
- Patients classified as polysensitized: demonstrated polysensitization by prick test results and/or singleplex specific IgE [sIgE] measurements.
- Patients selected for mutiplex testing by the allergy specialist. The criteria followed by the Allergy Service for multiplex IgE test prescription are as follows:
- Suspected LTP Syndrome in polysensitized patients to food and aeroallergens

**S2. Statistical methods**

The sensitization load for shared allergens was analyzed using a heatmap plot.

The association between ImmunoCAP and ALEX2 for tIgE was modeled using linear regression. Owing to the presence of large values (data not normally distributed), the model and scatter plot were based on log-transformed concentrations. The correlation between ImmunoCAP ISAC and ALEX2 was calculated using the Spearman coefficient and Bland Altman plotting for each allergenic component separately.

**S3. Clinical characterization. Clinical allergy triggers**

1. Nut allergy:

From the ten patients with clinical symptoms after nut consumption, the specific nut triggers were:

Patient 1: Peanut and walnut.

Patient 2: Hazelnut and almond.

Patient 6: Peanut

Patient 9: Peanut

Patient 13: Peanut and walnut

Patient 14: Walnut

Patient 15: Walnut and almond

Patient 17: Walnut

Patient 19: Hazelnut, almond and peanut.

Patient 20: Peanuts

1. Tomato allergy:

From the three patients showing tomato LTP sensitization, no one had specific clinical symptoms related to tomato in their clinical records.

Patient 2: General vegetables allergy, without a trigger identified in the clinical record.

Patient 3: No tomato allergy described in the clinical record.

Patient 14: No tomato allergy described in the clinical record.

1. Peach allergy:

From the 14 patients sensitized to Pru p 3, the following had a clinical diagnosis with peach allergy: 6, 14, 15, 17, 18, and 20. However, even some of them were not diagnosed with peach allergy, the clinical record clearly stated that, for example, the patients were eating peeled fruits or had allergy to vegetables, in general. So, probably, peach allergy was hidden in these unspecific comments from the clinical record.

1. LTP containing pollens allergy:

Patient 1: Plane tree and olive tree pollen

Patient 2: No respiratory allergy symptoms

Patient 3: No respiratory allergy symptoms

Patient 4: Olive tree pollen

Patient 5: Olive tree pollen

Patient 6: Olive tree pollen

Patient 7: Plane tree pollen and olive pollen

Patient 8: Olive tree pollen

Patient 9: Olive tree pollen

Patient 10: Seasonal asthma without trigger identified

Patient 11: Olive tree pollen

Patient 12: No respiratory allergy symptoms

Patient 13: Seasonal rhino conjunctivitis without trigger identified

Patient 14: Olive tree pollen

Patient 15: Olive tree pollen

Patient 16: Olive tree pollen

Patient 17: No respiratory allergy symptoms

Patient 18: Seasonal rhino conjunctivitis without trigger identified

Patient 19: Olive tree pollen

Patient 20: No respiratory allergy symptoms

**Figure S1.** Quantitative comparison (Spearman correlation) between ALEX2 and ISAC sensitization titers for each of the LTPs shared between the platforms. ALEX results are shown in kU_A_/L and ISAC results in ISU.


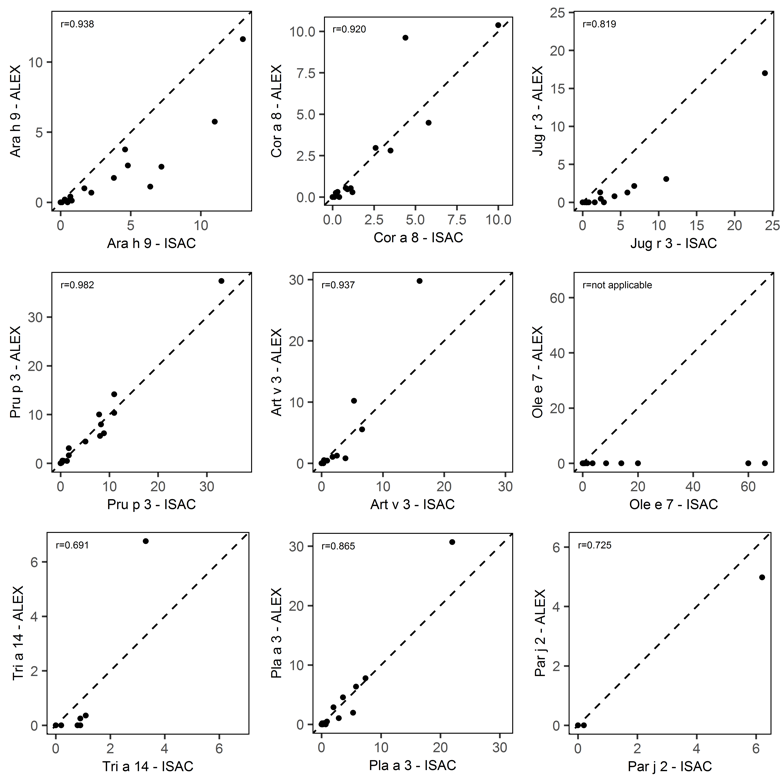


**Figure S2. A. Bland-Altman and B. Passing Bablock plots.** Comparison analysis between ALEX2 and ISAC sensitization titers for each of the LTPs shared between the platforms. Logarithmic scales were used in both cases.


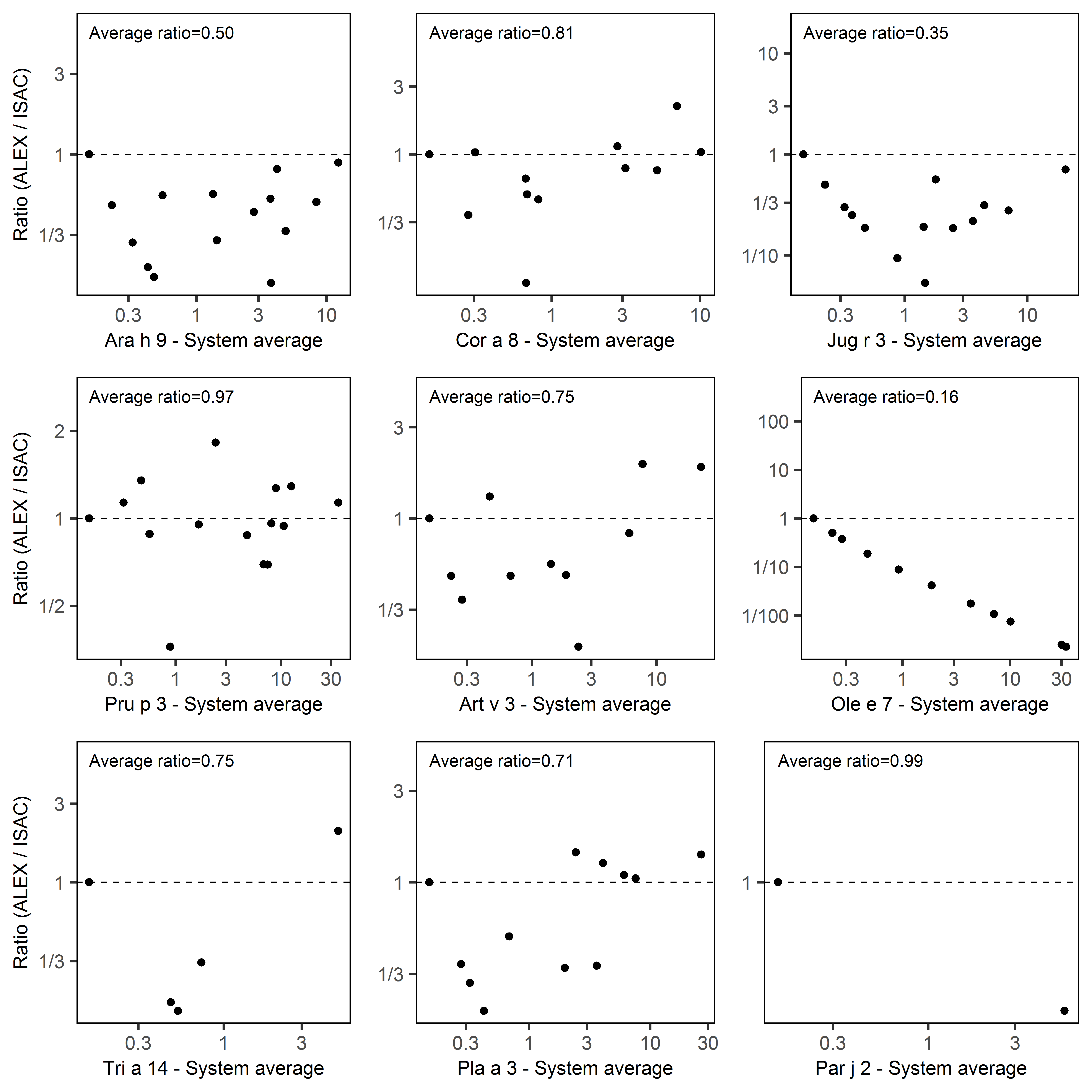

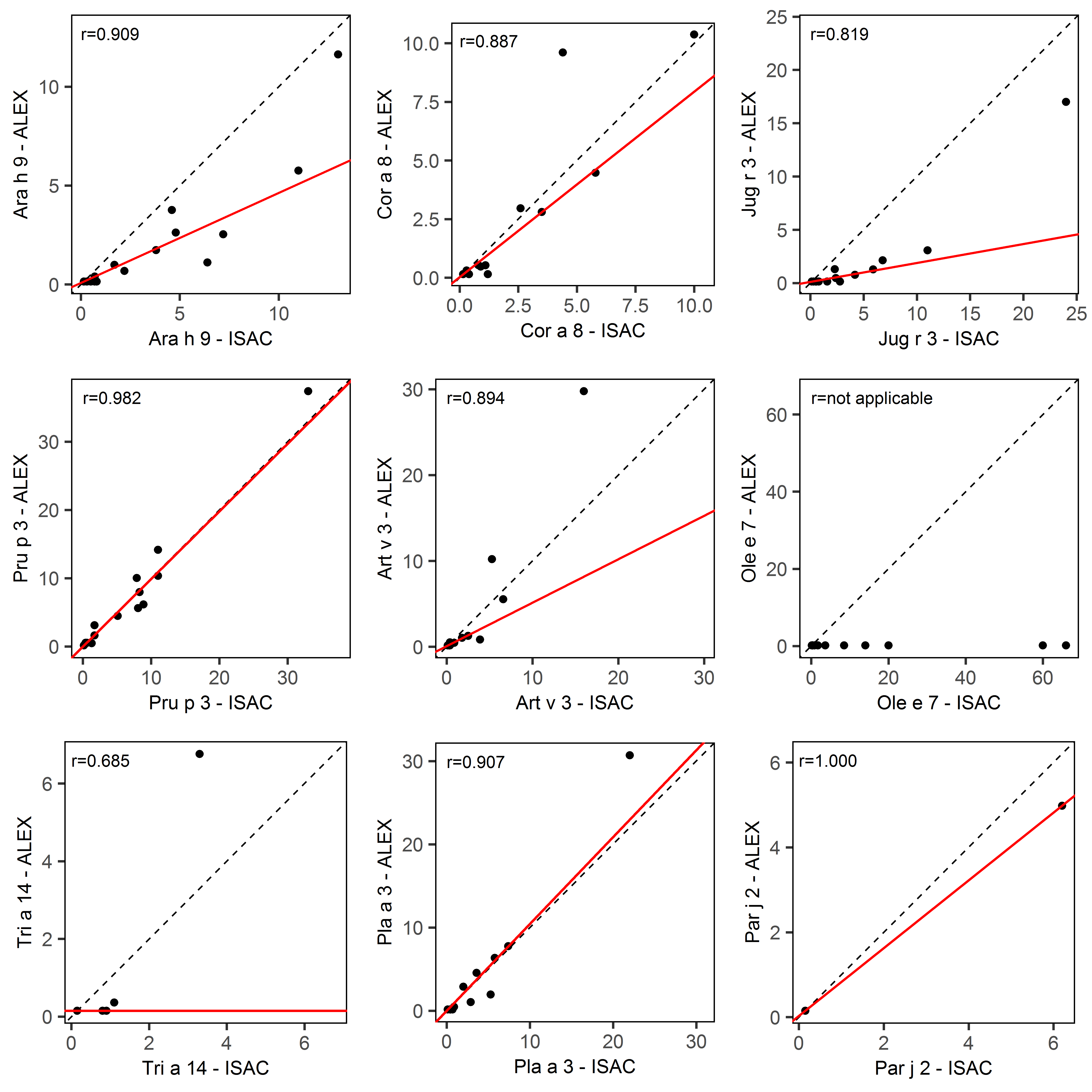


**A**

**B**

**Figure S3.** Total IgE regression study. Data are presented using a logarithmic scale. The broken black line shows perfect agreement. The red line shows the regression equation.


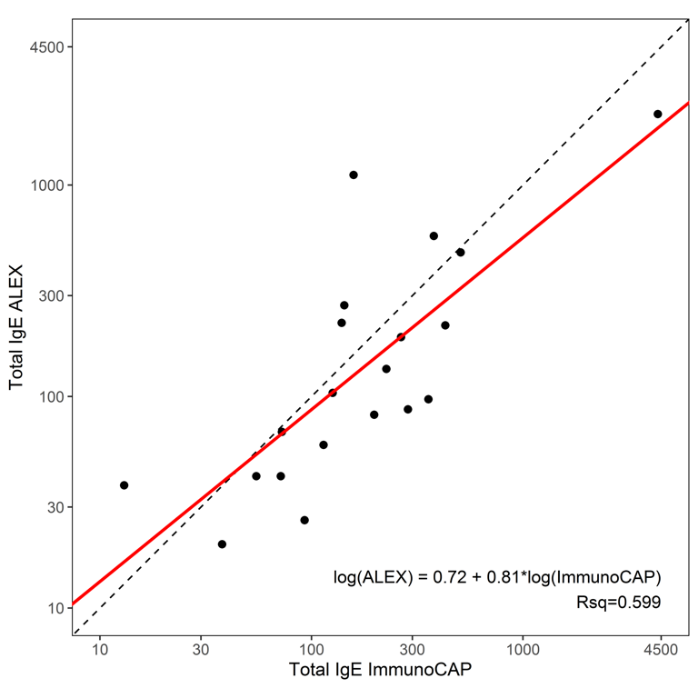


**Figure S4. A. Bland-Altman and B. Passing Bablock plots.** Comparison analysis between ALEX2 and ISAC tIgE. Logarithmic scales were used in both cases.


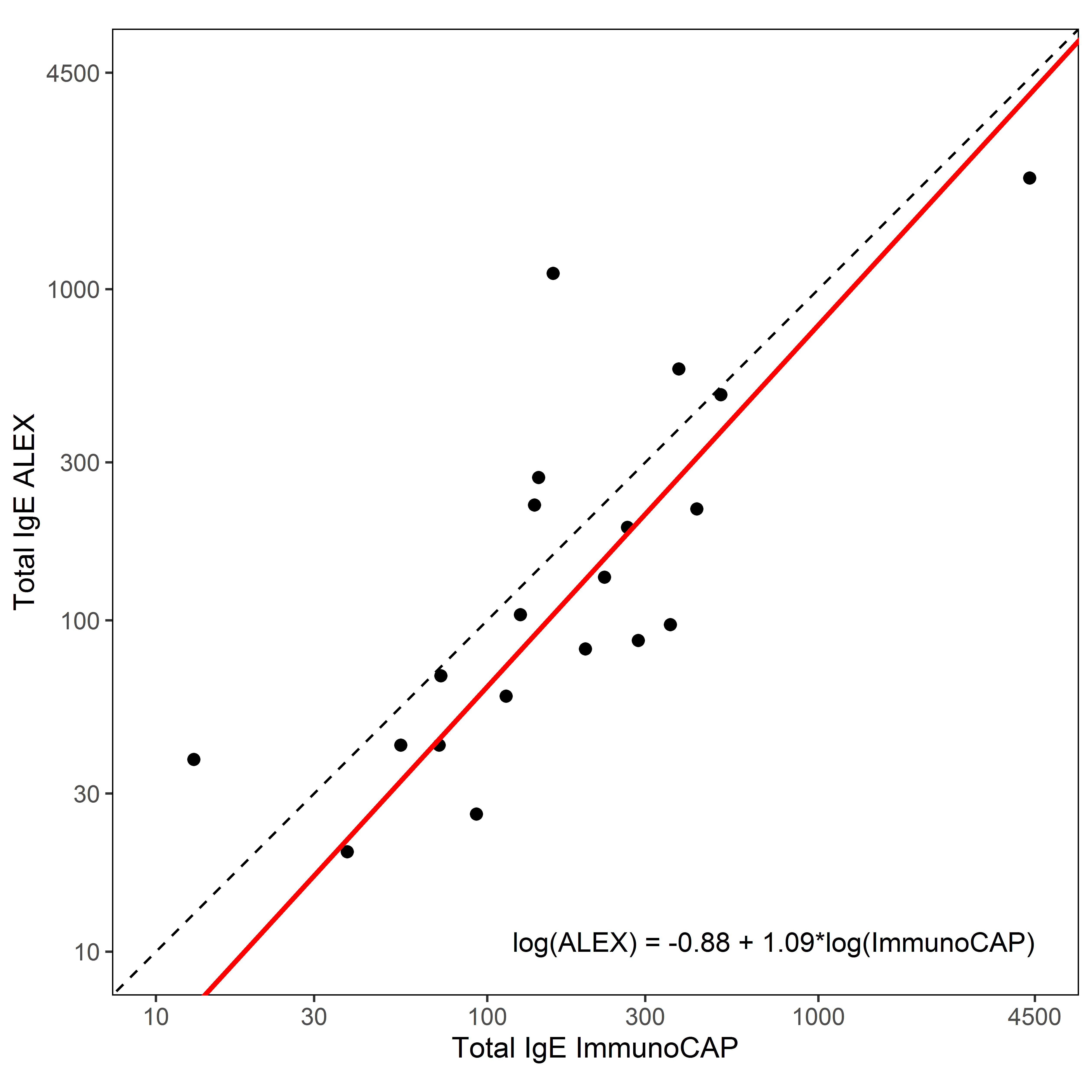


**B**


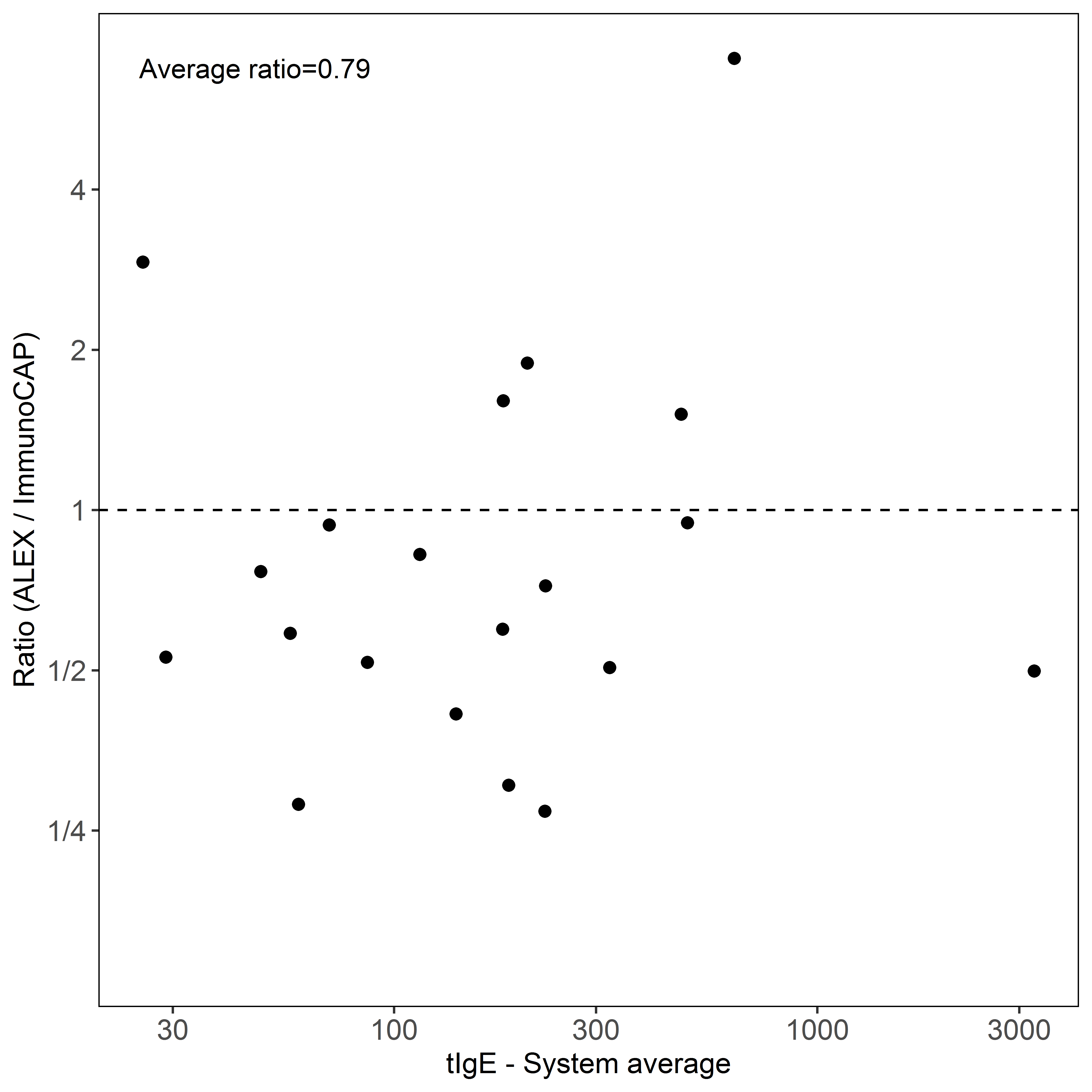


**A**

**Table S1.** Patient characteristics. Final diagnosis (food allergy, anaphylaxis, rhinitis and asthma) diagnosis was stablished according to clinical records. Mean total IgE was calculated using ImmunoCAP total IgE test results. Sensitization profiles were described according to ImmunoCAP ISAC and skin prick test results.

**Table S2a.** Sensitization values from both platforms for the LTPs. Total IgE values obtained by ImmunoCAP and ALEX2 are also shown for each patient. The Change (%) column includes the fold change for the same allergen from the same patient with both platforms. Green cells indicate determinations displaying higher results in ISAC. Red cells indicate determinations displaying higher results in ALEX.


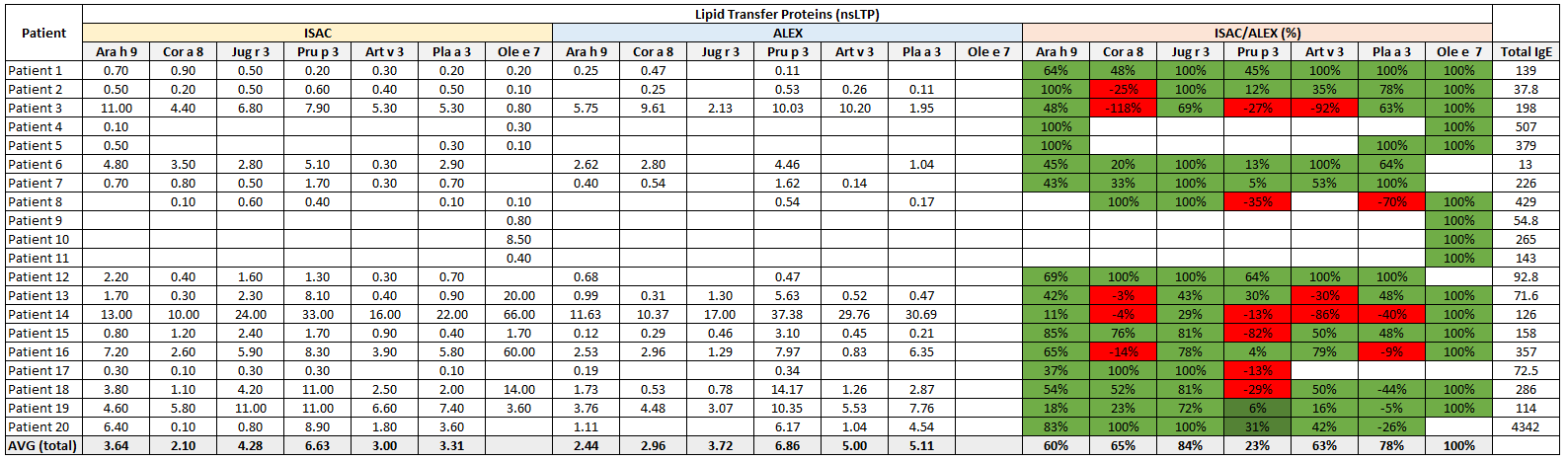


Table S2b. Sensitization values from both platforms for the clinically relevant LTPs. Total IgE values obtained by ImmunoCAP and ALEX2 are also shown for each patient. The Change (%) column includes the fold change for the same allergen from the same patient with both platforms. Green cells indicate determinations displaying higher results in ISAC. Red cells indicate determinations displaying higher results in ALEX.


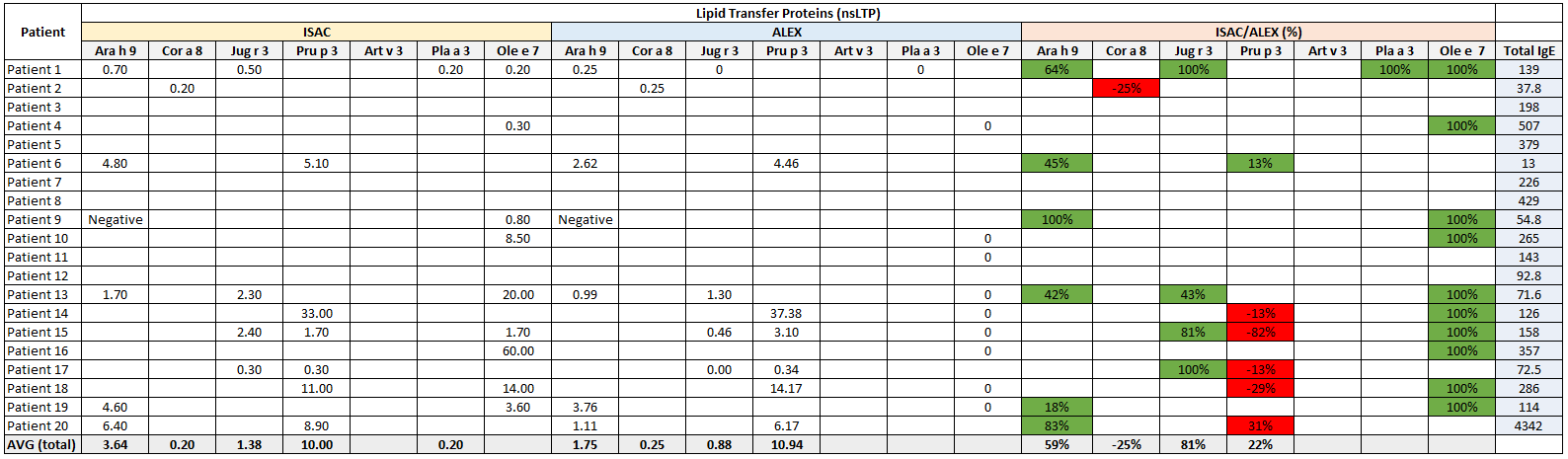


**Table S3.** Raw data corresponding to individual olive pollen component sensitization profiles.


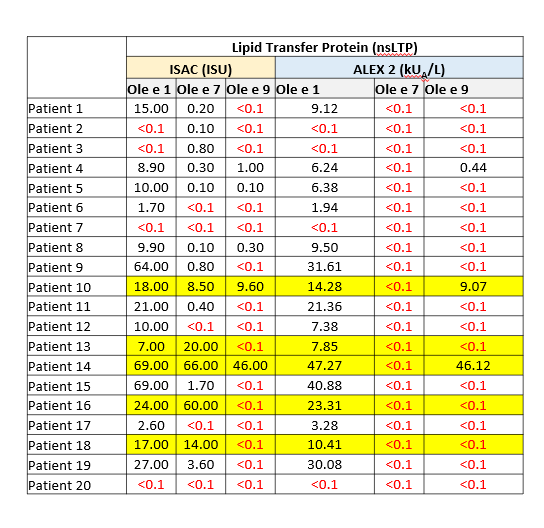


**Table S4.** sIgE values for *Solanum lycopersicum* whole extract and Sola l 6 (7kDa-LTP) component measured using ALEX2 in selected patients.


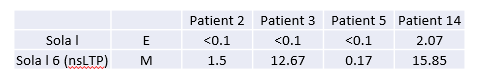

Supplement: Supplementary file 1 — Appendix S1. [file JCLA-37-e24960-s001.doc]
